# Beta-Actin Is Involved in Modulating Erythropoiesis during Development by Fine-Tuning Gata2 Expression Levels

**DOI:** 10.1371/journal.pone.0067855

**Published:** 2013-06-26

**Authors:** Davina Tondeleir, Benjamin Drogat, Karolina Slowicka, Karima Bakkali, Sonia Bartunkova, Steven Goossens, Jody J. Haigh, Christophe Ampe

**Affiliations:** 1 Department of Biochemistry, Faculty of Medicine and Health Sciences, Ghent University, Ghent, Belgium; 2 Vascular Cell Biology Unit, Department for Molecular Biomedical Research, VIB, Ghent, Belgium; 3 Department of Biomedical Molecular Biology, Ghent University, Ghent, Belgium; Baylor College of Medicine, United States of America

## Abstract

The functions of actin family members during development are poorly understood. To investigate the role of beta-actin in mammalian development, a beta-actin knockout mouse model was used. Homozygous beta-actin knockout mice are lethal at embryonic day (E)10.5. At E10.25 beta-actin knockout embryos are growth retarded and display a pale yolk sac and embryo proper that is suggestive of altered erythropoiesis. Here we report that lack of beta-actin resulted in a block of primitive and definitive hematopoietic development. Reduced levels of *Gata2*, were associated to this phenotype. Consistently, ChIP analysis revealed multiple binding sites for beta-actin in the *Gata2* promoter. *Gata2* mRNA levels were almost completely rescued by expression of an erythroid lineage restricted ROSA26-promotor based GATA2 transgene. As a result, erythroid differentiation was restored and the knockout embryos showed significant improvement in yolk sac and embryo vascularization. These results provide new molecular insights for a novel function of beta-actin in erythropoiesis by modulating the expression levels of *Gata2 in vivo*.

## Introduction

Actins are highly conserved proteins among various species throughout evolution [1]. The human genome has multiple functional actin genes and more than twenty pseudogenes [2], [3]. The expression patterns of vertebrate actins are temporally and spatially regulated during development and in the adult organism, suggesting different isoform specific functions [1], [4]. The four muscle-specific actins provide strength and contractility to muscle cells. By contrast beta- and gamma-cytoplasmic actins (further referred to as beta- and gamma-actin), that are coexpressed in all adult non-muscle tissues [5] are thought to participate in more dynamic actin cytoskeletal processes. Whereas gamma-actin seems to be uniformly distributed in all actin-containing structures, beta-actin seems to have a more polarized distribution, localized to the cortex of cells, specifically functioning in protrusive structures such as lamellipodia and filopodia [6], [7], [8], [9].

In more recent cell work, actin was found localized in the nucleus leading to the hypothesis that beta-actin could be implicated in modulating transcriptional activity. Actin interacts with all three RNA polymerases and beta-actin has been identified as a component of different types of chromatin remodeling complexes in a wide range of organisms [10]. In addition, antibodies against beta-actin block transcription [11] and nuclear translocation of beta-actin is involved in macrophage differentiation [12]. Although these previous studies obviously demonstrate a role for beta-actin in the nucleus, it is not clear if subsets of genes are dependent on beta-actin function in the nucleus and whether such mechanisms are important in instructing developmental processes.

A wealth of information regarding function and localization of actin isoforms was obtained from *in vitro* and *ex vitro* work, but studies on their role *in vivo* and in particular during mouse development, are lagging behind [13], [14]. The alpha-skeletal muscle actin knockout mouse and the alpha-cardiac muscle actin knockout mouse die, respectively, postnatally and perinatally due to muscle weakness [15], [16]. The alpha-smooth muscle actin knockout mice are viable but display cardiovascular defects [17]. Most gamma-actin knockout mice die within 48 hours after birth due to respiratory failure [18]. Mice that survive display progressive hearing loss. The embryonic lethality of the beta-actin knockout mice at E10.5, in spite of compensatory upregulation of other actins, suggests a lack of redundancy of actin functions at this stage in development [19]. In view of these observations, the functions of the cytoplasmic actins, and in particular of beta-actin, during development have remained poorly understood.

To contribute to knowledge on the function of beta-actin during mouse development we further investigated the possible causes of this early embryonic lethality by employing a heterozygous beta-actin knockout mouse that was previously generated (*Actb^+/−^*) [19]. We observed a diminished number of primitive erythroid cells and a paucity of well-organized blood islands that are the initial sites of primitive erythroid and endothelial cell development. We show that ablation of beta-actin expression during development interferes with red blood cell (RBC) development, resulting in reduced amounts of primitive and definitive erythroid cells in the *Actb^−/−^* embryos. Most strikingly, mRNA expression levels of *Gata2*, a transcription factor involved in early hematopoiesis, was dramatically decreased in yolk sacs of E8.5 *Actb^−/−^* embryos. Corroborating a role for beta-actin in *Gata2* regulation, we could show association of the beta-actin protein to the *Gata2* promoter. Further confirming a developmental link between beta-actin function and *Gata2* regulation, transgenic expression of *Gata2* specifically within the erythroid lineage in these *Actb^−/−^* embryos partially rescued the observed phenotypes. Our findings therefore support a novel function of beta-actin in modulating erythropoiesis by fine-tuning *Gata2* levels in the early developing mouse embryo.

## Materials and Methods

### Ethics Statement

The animal ethics committee of Ghent University approved all experiments performed on mice. Approval number ECD10/29.

### Mice

The generation of the *Actb^+/−^* mice has been previously described [19]. *Actb^+/−^* mice were crossed to generate control *Actb^+/+^* embryos and *Actb^−/−^* embryos. Genotyping was done by fluorescent microscopy (positive for *Actb^+/−^* and *Actb^−/−^*) followed by western blotting with an antibody against beta-actin (to distinguish *Actb^+/−^* and *Actb^−/−^*). Mice were kept on an inbred BALB/c background. *Actb^+/−^* mice were crossed with *Flk1*-LacZ mice [20] to generate double heterozygous knockouts. Genotyping was done by fluorescent microscopy followed by a LacZ PCR. *Actb^+/−^* mice were crossed with EpoR-iCre^Tg/+^ [21] mice and with ROSA26-hGata2^Tg/Tg^ [21] to generate the *Actb^−/−^* R26+hGata2^EpoR-iCre/+^ rescue mice. Genotyping was done by fluorescent microscopy followed by PCR.

### Antibodies

Antibodies (Ab) used in this study are rat-anti-mouse PECAM-1 mAb (Clone CD31), biotin-conjugated rat-anti-mouse PECAM-1 mAb (clone CD31) and biotin-conjugated goat-anti-rat Ig specific pAb, all from BD Pharmingen. ABC reagent (Vector Labs) was used with the biotin-conjugated CD31 mAb, whereas the Renaissance TSA Biotin System (PerkinElmer Life Sciences) was used with the other Abs. Anti beta-actin mAb (clone AC-15), from Sigma. Anti gamma-cytoplasmic actin pAb, from Millipore. Anti gata2 pAb, from Abcam.

### Paraffin histology

Dissected samples were fixed overnight in 4% paraformaldehyde at 4°C, processed for paraffin embedding, and sectioned at 6 µm. Sections were stained with hematoxylin and eosin (H&E). Additional paraffin sections were used for immunohistochemistry and immunofluorescence.

### Immunohistochemistry

Immunohistochemistry on whole mount embryos was performed as previously described [22]. Briefly, embryos were fixed in MeOH∶DMSO (4∶1) overnight at 4°C, treated with MeOH∶DMSO∶H_2_O_2_ (4∶1∶1) for 5–10 hours at room temperature to block endogenous peroxidase activity and stored in methanol at −20°C. The embryos were subsequently rehydrated in 50% MeOH in phosphate buffered saline PBS and incubated with the primary antibody in 3% instant skim milk powder/0.1% Triton X-100 in PBS (PBSMT) overnight at 4°C. Following washes in PBSMT for 5 hours at room temperature, embryos were incubated with the ABC reagent, in PBSMT overnight at 4°C. Following washes in PBSMT for 5 hours at room temperature and brief washes with PBS with 0.1% Triton X-100, the embryos were developed with 3.3-diaminobenzidine tetrahydrochloride (DAB) (Vector laboratories). The reaction was stopped by fixing the embryos in 4% PFA in PBS at room temperature for 1 hour. Immunohistochemistry on paraffin sections was done according to the protocol of the Renaissance TSA Biotin System (NEL 700, PerkinElmer). LacZ stainings were done according to manufacturer protocols (Millipore).

### Immunofluorescence

Immunofluorescence on paraffin sections started by deparaffinization through ethanol series. Sections were microwaved in 0,01 M citrate buffer (pH 6.0) for 15 min at full power and washed in PBS. Sections were blocked in 10% goat serum/1% BSA in PBS for 1 hour at room temperature and incubated with primary antibody in blocking solution overnight at 4°C. Following four washes with PBS of each 30 min at room temperature, sections were incubated with secondary antibody for 2 hours at room temperature. Following four washes with PBS of each 15 min at room temperature, nuclei of cells were stained with DAPI and sections were mounted with DABCO mounting medium (Sigma-Aldrich).

### Molecular analysis

Yolk Sacs (YS) were dissected at E8.5 and E10.25, or in case of *Actb^−/−^* R26+hGata2^EpoR-iCre/+^ at E10.25 and E11.5, flash-frozen in liquid nitrogen, and processed according to standard protocols. RNA was extracted with High Pure RNA isolation kit (Roche) and precipitated overnight at −80°C or 1 hr on dry ice. cDNA was synthesized with the transcriptor first strand cDNA synthesis kit (Roche). Quantitative reverse-transcription-polymerase chain reaction (qRT-PCR) was performed on a LightCycler 480 system (Roche) using the SYBR Green Master kit (Roche). Gene expression was normalized using glyceraldehyde 3-phosphate dehydrogenase (*Gapdh*), ubiquitine C (*Ubc*), glucose-6-phosphate dehydrogenase, (*G6pdh*) and hypoxanthin phosphoribosyl transferase 1 (*Hprt1*) as controls. Primers used are given in Table S1. The expression levels of each gene are reported relative to those observed in *Actb^+/+^* control samples. For sampling and statistics see below.

### ChIP

Bone marrow cells were cross-linked in 1% formaldehyde for 10 minutes at room temperature and the reaction was stopped with glycine (final concentration 0,125 M). Cells were lysed in the presence of protease inhibitors and DNA was sonicated. For each immunoprecipitation, 100 µg DNA was used together with 3 µl of anti beta-actin mAb (clone AC-15, Sigma) or anti gamma-cytoplasmic actin pAb (Millipore). Complexes were precipitated with protein A and G Sepharose beads (GE Healthcare). Formaldehyde cross-links were reversed by overnight incubation at 65°C and DNA was purified using QIAquick PCR Purification Kit (Qiagen). Matinspector was used to analyse amplicons for DNA core consensus sites. Primers used are given in Table S2, including localization of the respectively amplicons on the *Gata2* promotor.

### In vitro hematopoietic progenitor assays

For primitive erythroid (EryP) colony assays were performed on stage matched E8.5 whole embryos as previously described [21]. Definitive erythroid colony assays were performed on E9.75 yolk sacs as previously described [21] using 1% methylcellulose (StemCell Technologies) containing complete recombinant cytokines (MethoCult GF M3434) for the detection and quantification of burst forming unit-erythroid cells (BFU-E), colony forming unit-granulocyte/macrophage (CFU-GM) and colony forming unit-granulocyte/erythrocyte/monocyte/megakaryocyte (CFU-GEMM). After 7 days colonies were scored under a microscope. The results are expressed as a percentage of absolute number of colonies per yolk sac of the *Actb^+/+^* controls.

### Imaging

Embryos were imaged on a Leica MS5 (Leica Microsystems) stereomicroscope. Digital images were acquired using a Leica camera. Section H&E staining and immunohistochemistry were imaged using a SNAP-COOL camera (Roper Scientific) mounted on an Olympus Bx51 microscope (Olympus), with Plan Olympus 20×/0.40 or 40×/0.65 lens and RSImage Version 1.9.2 software (Roper Scientific).

### Statistical analysis

Data were expressed as mean plus or minus SEM. Comparison between 2 data groups was done by the 2-sided Student t test. A minimum of three biological replicates was used in each condition for each genotype. Two technical replicates were used per biological replicate.

## Results

### Yolk sac blood island development is affected in beta-actin knockout embryos

**Figure 1:**
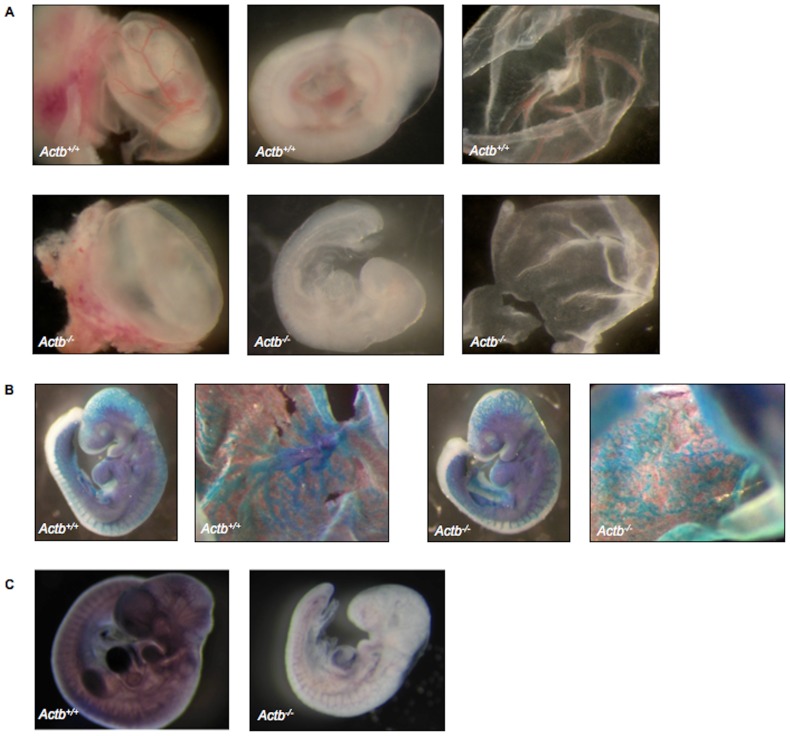
Actb−/− embryos are pale and growth retarded at E10.25. (A) Pictures of freshly dissected embryos at E10.25. Pale and growth retarded *Actb^−/−^* embryos show no obvious vascular pattern in the embryo proper (middle) or yolk sac (right) compared to the *Actb^+/+^* littermates. 15× magnification. (B) LacZ stainings of *Actb^+/+^* and *Actb^−/−^* yolk sac at E9.5 indicate reduced vascular branching complexity of *Actb^−/−^* yolk sacs. 20× magnification. (C) Whole mount PECAM-1 immunohistochemistry of *Actb^+/+^* and *Actb^−/−^* embryos, processed in parallel, shows less coloring, indicating fewer endothelial cells and red blood cells. 15× magnification. Embryos were imaged on a Leica MS5 (Leica Microsystems) stereomicroscope. Digital images were acquired using a Leica camera.

To specifically address the role of beta-actin in development, a beta-actin knockout mouse allele was used (*Actb^+/−^*). It was previously shown that the homozygous beta-actin knockout mice (*Actb^−/−^*) are lethal at E10.5 [19], however the exact cause of lethality has not been investigated. At E8.5, no obvious morphological differences between *Actb^+/+^* and *Actb^−/−^* embryos could be observed (data not shown). One day later at E9.5 *Actb^−/−^* embryos have an appropriate number of somites and were in that aspect comparable in development to *Actb^+/+^* littermates. However, the *Actb^−/−^* embryos display a visibly pale yolk sac and embryo proper (Figure 1A). Yolk sacs of *Actb^+/+^* embryos displayed large RBC containing vessels, whereas no such vessels could be observed in *Actb^−/−^* yolk sacs. (Figure 1A). To study the branching pattern of the vasculature in the yolk sac, *Actb^+/−^* mice were crossed with *Flk1*-LacZ mice [20]. *Flk1* marks vascular and hematopoietic progenitors and the *Flk1*-LacZ mice express a LacZ reporter under control of the *Flk1* promotor. It is therefore possible to monitor the migration of hemangioblast progenitors from the primitive streak towards the yolk sac and to study the vasculogenesis process. The resulting E9.5 *Actb^−/−^ Flk1*-LacZ embryos displayed clearly reduced vascular branching complexity in the yolk sac and specifically in the head region of the embryo proper compared to the *Actb^+/+^ Flk1*-LacZ littermates (Figure 1B). Especially the remodeled large branched vessels, which are already prominent in yolk sacs of *Actb^+/+^ Flk1*-LacZ embryos seemed to be absent in the *Actb^−/−^ Flk1*-LacZ embryos. Rather the *Actb^−/−^* yolk sacs still contained the honeycomb-shaped capillary plexus (typical for E8.5 embryos), which did not reorganize into large branches, suggesting a block in proper vasculo/angiogenesis development.

**Figure 2:**
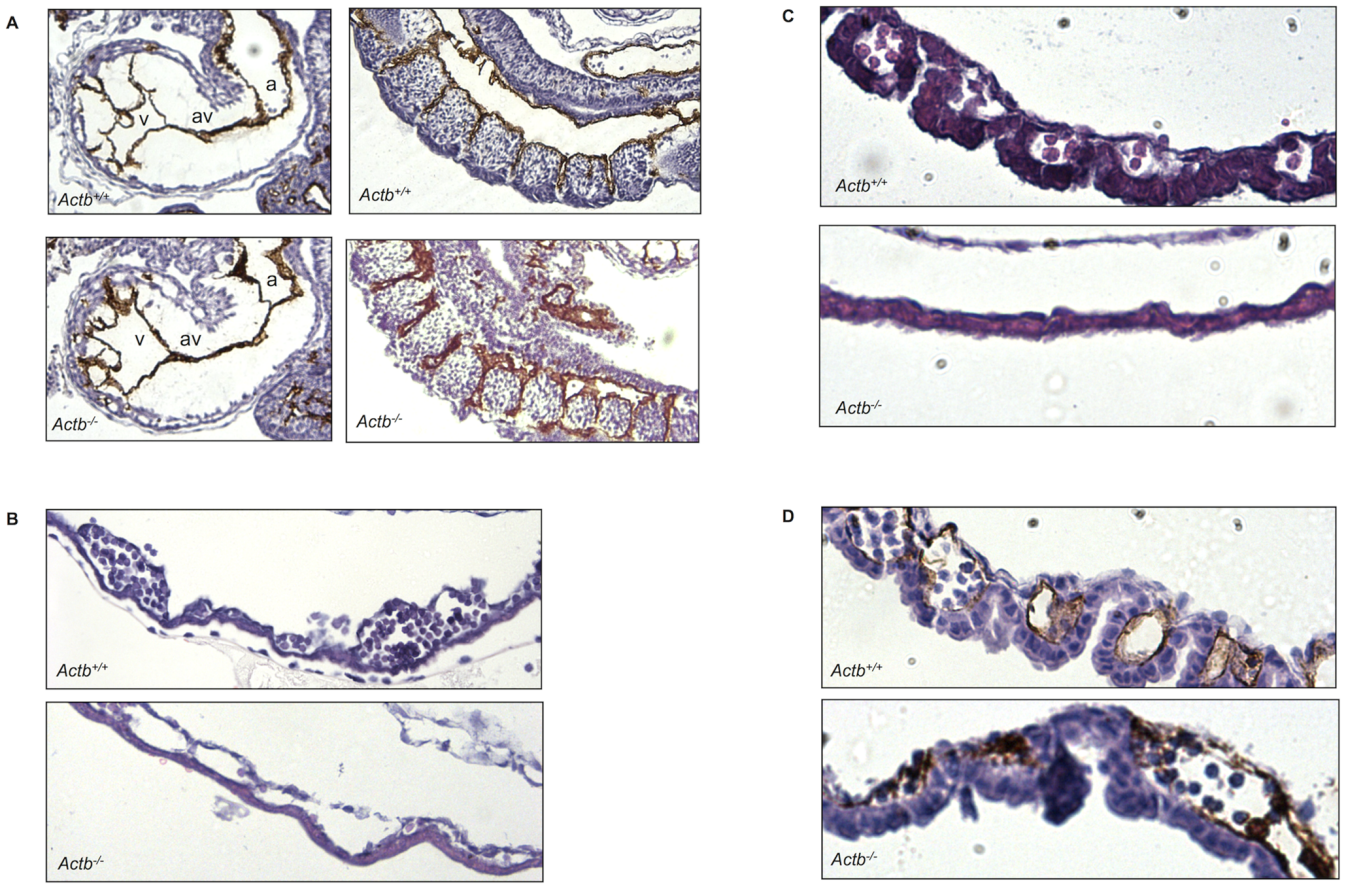
Actb−/− embryos show reduced number of red blood cells and abnormal blood island morphology in the yolk sac. (A) PECAM-1 immunostained sagittal cardiac and somitic section, showing normal development of the left atrium (a), atrioventricular canal (av), left ventricle (v) and regular development of intersomitic vessels. (B) H&E stained yolk sac sections show disrupted blood island morphology with empty, enlarged cavities in E9.5 *Actb^−/−^* embryos. A and B at 20× magnification. (C) H&E stained yolk sac sections show an almost complete absence of blood islands in E10.25 *Actb^−/−^* embryos. 40× magnification. (D) PECAM-1 immunostained yolk sac section showing disorganized endothelial patterning and almost no red blood cells populating the remaining blood islands at E10.25. C and D at 40× magnification. Sections were imaged using a SNAP-COOL camera (Roper Scientific) mounted on an Olympus Bx51 microscope (Olympus), with Plan Olympus 20×/0.40 or 40×/0.65 lens and RSImage Version 1.9.2 software (Roper Scientific).

At E10.25, the *Actb^−/−^* embryos were morphologically growth retarded. In view of the impaired vascular development of the yolk sac at E9.5 we performed PECAM-1 whole mount immunostaining at E10.25 embryos. This analysis showed the presence of a more weakly stained vasculature and therefore suggests diminished numbers of endothelial cells in *Actb^−/−^* embryos, similar to the yolk sac vascular defects (Figure 1C). PECAM-1 immunostaining on sections from E9.5 embryos demonstrated normal endocardial and slightly abnormal intersomitic vessel development (Figure 2A and data not shown).

More detailed analysis of yolk sacs of *Actb^−/−^* embryos at E9.5 revealed enlarged blood islands harboring fewer RBCs compared to wild type *Actb^+/+^* yolk sacs (Figure 2B). At E10.25, routine H&E staining and PECAM-1 immunohistochemistry revealed abnormal vessel morphology and endothelial patterning in *Actb^−/−^* yolk sacs (Figure 2C-D). We conclude that blood island morphology is largely impaired.

### Absence of beta-actin modulates yolk sac erythropoiesis

**Figure 3:**
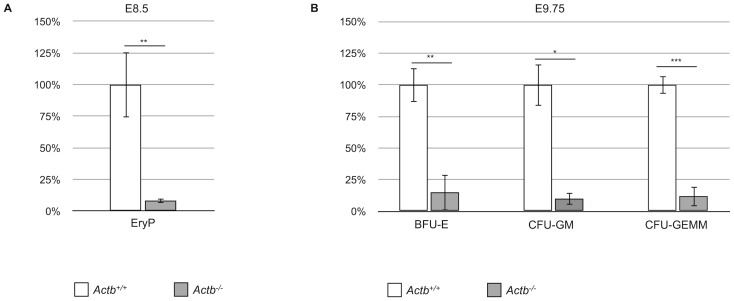
Yolk sac erythropoiesis is impaired in Actb−/− embryos. (A) Primitive erythroid colonies (EryP) from E8.5 yolk sacs measured by methylcellulose assays show a dramatic decrease in colony forming potential in *Actb^−/−^* embryos compared to *Actb^+/+^* embryos. Results are given as percentage of *Actb^+/+^* embryos absolute number of colonies (100%). (B) Definitive erythroid colonies (BFU-E, CFU-GM and CFU-GEMM) from E9.75 yolk sacs measured by methylcellulose assays show an 85 to 90% decrease in colony forming potential of *Actb^−/−^* embryos versus *Actb^+/+^* embryos. Results are given as percentage of *Actb^+/+^* embryos absolute number of colonies (100%). Bars represent mean ±SEM; *P<.05, **P<.01, ***P<.001.

As the embryo becomes larger, diffusion of oxygen is no longer sufficient at E9.5. To accommodate the embryo proper with sufficient oxygen, primitive erythropoiesis takes place before this stage, enabling the production of a temporary wave of primitive RBCs in the blood islands of the yolk sac. In view of the pale yolk sacs of *Actb^−/−^* embryos, suggesting reduced numbers of primitive RBCs, we investigated if primitive erythropoiesis was affected. Although there was no difference in the morphology of erythroid progenitor (EryP) colonies, the number of colonies derived from E8.5 *Actb^−/−^* embryos was decreased by more than 90% compared to *Actb^+/+^* embryos (Figure 3A), a result that is consistent with the low amount of RBCs observed in freshly dissected embryos and sections (Figure 1–2).

The initial phase of primitive erythropoiesis is succeeded by definitive embryonic erythropoiesis, and therefore we also quantified definitive erythroid progenitors *in vitro*. Methylcellulose assays conducted on E9.75 yolk sacs showed an 85% decrease of BFU-E and 90% decrease of CFU-GM and CFU-GEMM colony numbers in *Actb^−/−^* embryos (Figure 3B). Again, the colonies formed in methylcellulose did not morphologically differ from the colonies of *Actb^+/+^* littermates. These results demonstrate that absence of beta-actin has negative effects on embryonic hematopoiesis.

### Absence of primitive erythropoiesis in *Actb^−/−^* embryos correlates with reduced *Gata2* expression

**Figure 4:**
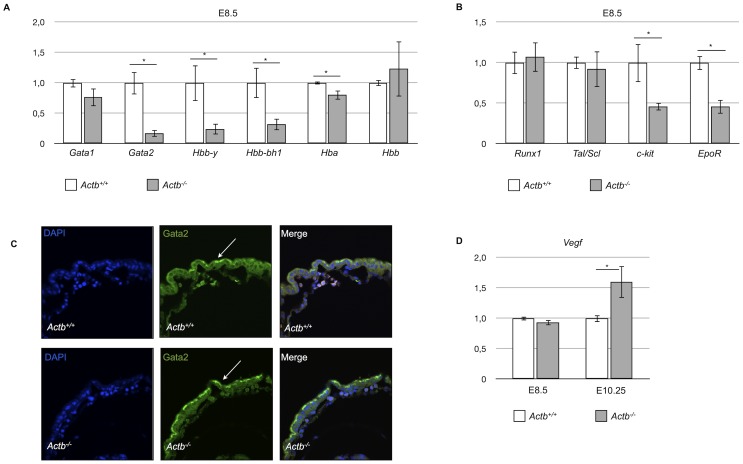
Absence of beta-actin during primitive erythropoiesis correlates with reduced Gata2 expression levels in the yolk sac. (A) Relative E8.5 yolk sac mRNA levels measured by qRT-PCR of *Gata1*, *Gata2*, Hbb-y, Hbb-bh1, Hba and Hbb. We found a 90% decrease of *Gata2* expression level in *Actb−/−* embryos versus *Actb+/+* embryos. (B) Relative E8.5 yolk sac mRNA levels measured by qRT-PCR of *Gata2* target genes: *Runx1*, Tal1/Scl, *c-kit* and *EpoR*. *c-kit* and *EpoR* show 50% reduced expression levels in *Actb−/−* embryos versus *Actb+/+* embryos. (C) *Gata2* immunohistochemistry on E9.5 yolk sac sections. *Gata2* expression was detected only in the endoderm of the yolk sac (white arrows). No difference in *Gata2* expression could be seen between *Actb−/−* embryos and *Actb+/+* embryos. 60× magnification. (D) Relative E8.5 and E10.25 yolk sac mRNA levels measured by qRT-PCR of Vegf. At E10.25, we could demonstrate a 50% increase of Vegf mRNA expression. Error bars represent mean ±SEM; *P<.05. Immunofluorescence sections were imaged using an Olympus IX81 confocal microscope with Fluoview FV10 software.

To correlate these effects on embryonic erythropoiesis in the *Actb^−/−^* embryos with molecular alterations of known transcriptional modulators that play important roles in erythropoiesis, we performed qRT-PCR mRNA expression analysis on *Actb^−/−^* versus *Actb^+/+^* yolk sacs for key erythroid transcription factors *Gata1* and *Gata2*. Since separation of endothelial and hematopoietic cells from yolk sacs of this stage is technically extremely challenging, we used the whole yolk sac. We also tested the embryonic globins Hbb-y and Hbb-bh1 and the adult globins Hba and Hbb. At E8.5, no morphological differences could be observed between *Actb^−/−^* and *Actb^+/+^* embryos. We found no significant changes for *Gata1* and Hbb. Hba gave a slight but significant reduction (Figure 4A). However, the most dramatic effects were seen for *Gata2*, Hbb-y and Hbb-bh1 expression. *Gata2* was decreased by 90% and Hbb-y and Hbb-bh1 expression levels were decreased by respectively 75% and 70% (Figure 4A). This further corroborates the negative effects of beta-actin depletion on primitive erythropoiesis observed in our methylcellulose experiments. Our results regarding the observed decrease in primitive hematopoiesis in the *Actb^−/−^* embryo are consistent with the observed anemia and lethality at E10.5 in *Gata2* knockout embryos [23] indicating that the diminished expression of *Gata2* contributes to this phenotype in the *Actb^−/−^* embryos.

We also quantified target genes of *Gata2* at E8.5: *Runx1*, *Tal/Scl*, *c-kit*, *EpoR*, *Hhex* and *Lmo2* (Figure 4B). In none of the genotypes we detected expression of *Hhex* and *Lmo2* at this developmental stage (data not shown). *Runx1* and *Tal/Scl* expression levels gave no significant differences but *c-kit* and *EpoR* were downregulated by more than 50% in *Actb^−/−^* yolk sacs, further emphasizing the consequences of *Gata2* downregulation in the *Actb^−/−^* embryos.

*Gata2* is also expressed in the vascular endothelium of the embryo proper [24]. To differentiate between the remodeling defects of yolk sac blood vessels versus the impaired erythropoiesis, we studied the expression of GATA2 protein in the blood islands at E9.5. In the *Actb^+/+^* embryos GATA2 protein is expressed in primitive erythrocytes and to a moderate extent in the yolk sac endoderm. However, no GATA2 expression could be seen in the vascular endothelial cells of the yolk sac. Importantly, we could not detect a difference between GATA2 expression in the endoderm nor in the vascular endothelial cells of *Actb^−/−^* versus the *Actb+/+* yolk sacs, suggesting that the vascular remodeling defect is not caused by a differential expression of GATA2 (Figure 4C), but rather by an impaired blood flow and/or increased hypoxia due to lack of erythroid differentiation. Indeed, we also tested Vegf expression levels in the yolk sac at E8.5 and E10.25 (Figure 4D). At E10.25, Vegf mRNA levels are more than 50% higher in *Actb^−/−^* yolk sacs versus *Actb+/+* yolk sacs, suggesting hypoxic conditions in the embryos. Moreover, the *Gata2* target gene *Tal/Scl*, involved in the development of the vascular endothelium [24], shows no difference in expression between *Actb+/+* and *Actb^−/−^* yolk sacs (Figure 4B), further supporting the hypothesis that impaired blood flow or hypoxic insult arising due to a lack of erythropoiesis is causing the vascular remodeling defects.

### Compensatory up regulation of gamma-actin does not prevent lethality

In *Actb^−/−^* embryos as well as in *Actb^−/−^* mouse embryonic fibroblasts (MEFs) and T-cells, gamma-actin, the second cytoplasmic isoform, is upregulated [13], [18]. To investigate whether this is also the case in *Actb^−/−^* yolk sacs we compared gamma-actin expression levels with *Actb^+/+^* control yolk sacs by qRT-PCR and Western blot analysis. At E8.5, there is an approximate two-fold increase of gamma-actin mRNA expression and only a slight increase of gamma-actin protein in the *Actb^−/−^* versus *Actb^+/+^* yolk sacs (Fig S1A–B). At E10.25, gamma-actin mRNA expression patterns remained doubled and the gamma-actin protein levels were increased compared to the E8.5 timepoint (Fig S1A–C). In view of the observation that gamma-actin is the dominant isoform during mouse organogenesis (Fig S1D), this continuing compensatory expression of gamma-actin in the *Actb^−/−^* yolk sac is apparently not capable of rescuing the hematopoietic related phenotypes and indicates that the functions of both cytoplasmic isoforms are in this process non redundant.

### Beta-actin linked to several regions of the *Gata2* gene

**Figure 5:**
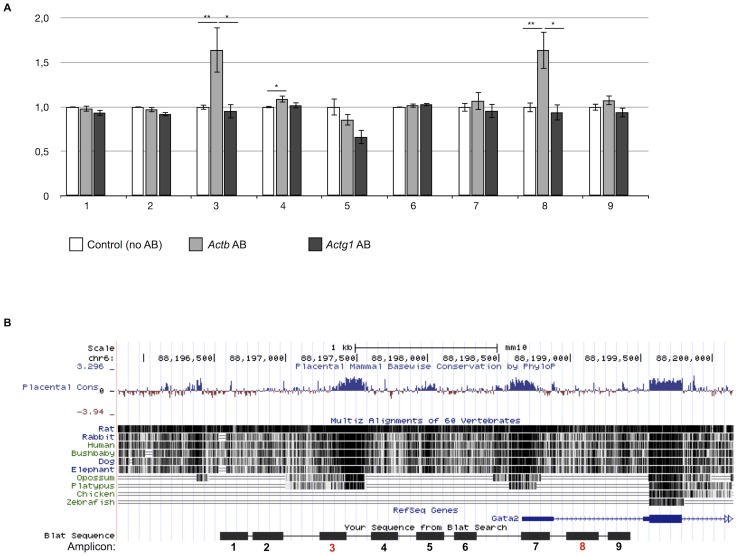
Beta-actin binds to specific regions of the *Gata2* gene. (A) Immunoprecipitation with the anti beta-actin and anti gamma-actin antibodies followed by qRT-PCR with 9 specific primers for the *Gata2* promotor region yielded 2 loci of interest: amplicon 3 and 8. Error bars represent mean ±SEM; *P<.05, **P<.01. (B) Genomic alignment with multiple species showing that amplicon 3 is partly overlapping with a highly conserved region in the *Gata2* promotor, especially in mammals. Also the localization of the specific amplicons relative to the mouse *Gata2* gene is shown. Amplicon8 is located between exons 1 and 2. Figure was made using UCSC genome bioinformatics software (http://genome.ucsc.edu/).

To begin determining the molecular basis linking beta-actin to *Gata2* expression, we performed ChIP analysis. Since it is technically impossible to obtain enough hematopoietic cells from these embryos to perform ChIP experiments, we isolated bone marrow cells from wild type mice. We used 9 primer sets covering 3 kbp of the *Gata2* promotor region. As shown in Figure 5A, immunoprecipitation with the anti beta-actin antibody followed by qRT-PCR with specific primers for the *Gata2* promotor region yielded two loci of interest (amplicon 3 and 8, Figure 5A), indicating that beta-actin binds directly or indirectly to the *Gata2* promotor. In contrast, immunoprecipitation with an antibody against gamma-actin did not yield any amplicon signal, indicating that beta-actin specifically binds to these regions of the *Gata2* promotor.

Genomic alignment of these amplicon sequences using BLAT (http://genome.ucsc.edu/) indicated that amplicon 3 is partly overlapping with a highly conserved region within the *Gata2* promotor (Figure 5B). The fact that this domain is highly conserved during mammalian evolution suggest that it may contain important regulatory binding sites that control *Gata2* transcription. Therefore, we screened the amplicon 3 sequence as well as flanking sequences for putative core consensus binding sites of transcription factors playing key roles in hematopoiesis and blood island formation. Multiple DNA core consensus sites were found (Figure S2) such as target binding sequences for *Runx1*, *Hhex*, Szf1, Mzf1 and *Tal/Scl* [25], [26], [27], [28] transcription factors with a role in hematopoiesis. Contained within these conserved sequences we also found binding sites for the *CP2*, *Klf1* (*Eklf*) and *Zbp-89* transcription factors that are specifically involved in the regulation and/or maturation of erythroid progenitors [29], [30], [31]. Other putative sites found within these regions are for transcription factors that are downstream effectors of multiple signaling pathways (*NF-kB* and *c-Rel*) [32], functioning in hypoxia signaling cascades (*Hif-1*) [33] or functioning as chromatin organizers (*Ctcf*) [34]. Interestingly, we also identified a site for *ZF161* (*ZF5*), an activator or repressor of transcription that was shown to repress the Actb promotor [35]. Amplicon 8 harbors a reverse CCAAT box, the consensus sequence for the maternal CCAAT box transcription factor, specifically necessary for full promotor activity of the *Gata2* gene [36].

### Erythroid restricted ROSA26-promoter based expression of *Gata2* partially rescues erythropoiesis block of *Actb^−/−^* embryos

**Figure 6:**
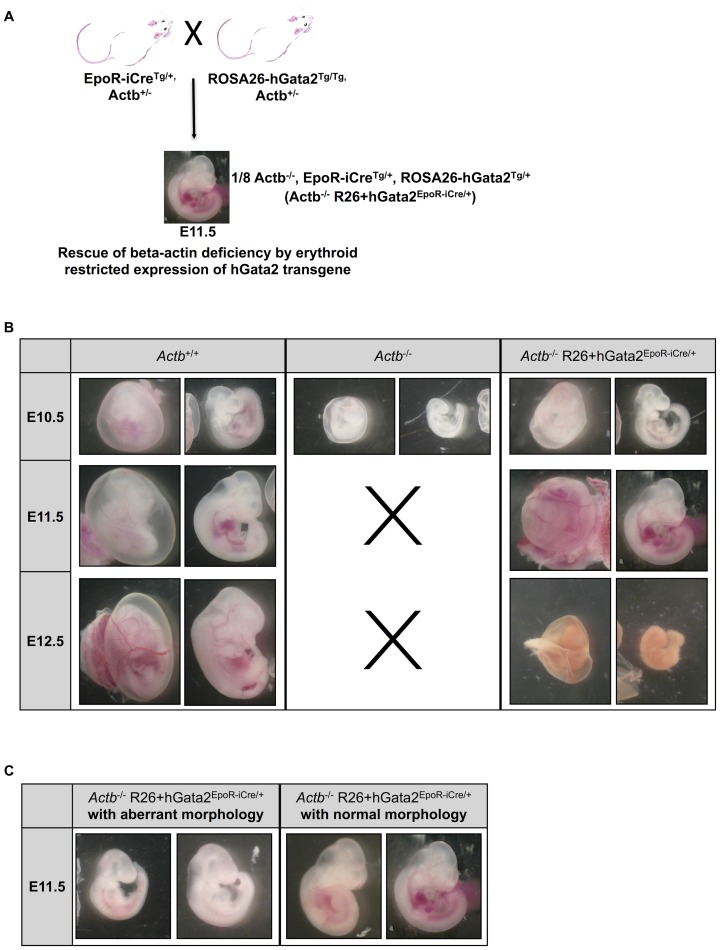
*Actb−/−* R26+hGata2EpoR-iCre/+ embryos remain viable at E11.5. (A) Schematic view of the breeding scheme used to generate *Gata2* expression in the erythroid lineage in *Actb−/−* mice. (B) Pictures from freshly dissected embryos at developmental stages E10.5, E11.5 and E12.5. Black crosses mark the absence of *Actb−/−* embryos at stages E11.5 and E12.5. Note the presence of RBCs in the *Actb−/−* R26+hGata2EpoR-iCre/+ embryos. Statistics on embryo recovery in function of genotype are in Table S3. E10.5: 15× magnification, E11.5: 12× magnification; E12.5: 10× magnification. (C) Morphology variation of *Actb−/−* R26+hGata2EpoR-iCre/+ embryos at E11.5. Aberrant looking *Actb−/−* R26+hGata2EpoR-iCre/+ embryos (left panel) compared to normal looking *Actb−/−* R26+hGata2EpoR-iCre/+ embryos (right panel), 12× magnification. Embryos were imaged on a Leica MS5 (Leica Microsystems) stereomicroscope. Digital images were acquired using a Leica camera.

**Figure 7:**
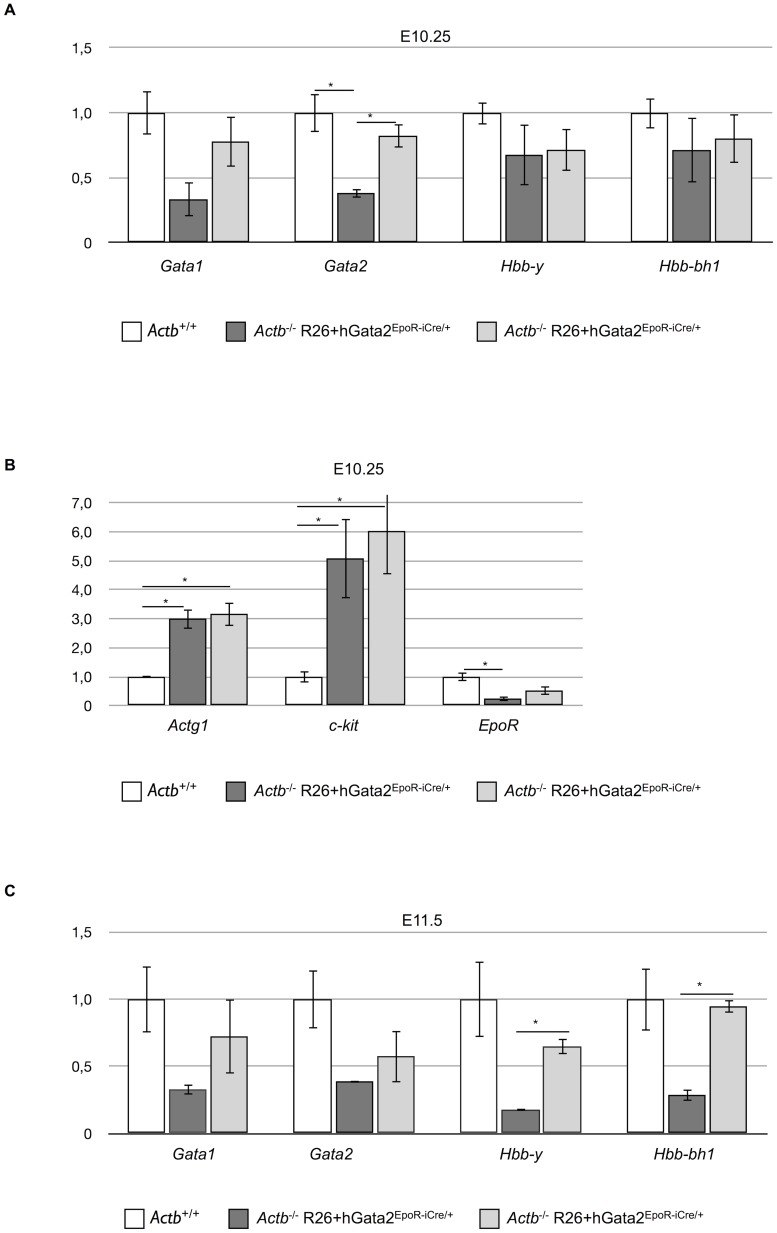
Morphologically normal *Actb−/−* R26+hGata2EpoR-iCre/+ embryos show improved Gata2 mRNA levels compared to *Actb−/−* embryos. (A) Relative E10.25 mRNA levels measured by qRT-PCR of *Gata1*, *Gata2*, Hbb-y, and Hbb-bh1 in yolk sacs of *Actb+/+*, *Actb−/−* R26+hGata2EpoR-iCre/+ embryos with aberrant morphology and *Actb−/−* R26+hGata2EpoR-iCre/+ embryos with normal morphology. (B) Relative E10.25 mRNA levels measured by qRT-PCR of Actg1 and *Gata2* targets: *c-kit*, and *EpoR* in yolk sacs of *Actb+/+*, *Actb−/−* and the two groups of *Actb−/−* R26+hGata2EpoR-iCre/+ embryos. (C) Relative E11.5 mRNA levels measured by qRT-PCR of *Gata1*, *Gata2*, Hbb-y, and Hbb-bh1 in yolk sacs of *Actb+/+*, *Actb−/−* R26+hGata2EpoR-iCre/+ embryos with aberrant morphology (see Figure 6C) and *Actb−/−* R26+hGata2EpoR-iCre/+ with normal morphology. White bars  = *Actb+/+*, dark-grey bars  = *Actb−/−* R26+hGata2EpoR-iCre/+ with aberrant morphology, light-grey bars  = *Actb−/−* R26+hGata2EpoR-iCre/+ with normal morphology.

To further confirm the functional involvement of decreased *Gata2* levels in the erythropoiesis block in *Actb^−/−^* embryos and to demonstrate that the vascular defects are secondary to this block in erythroid differentiation we attempted to rescue the lethality using erythroid lineage restricted expression of a ROSA26-promoter based transgenic expression of human Gata2 (hGata2). The erythroid lineage restricted expression of Gata2 was previous described in literature [21], [37]. We generated EpoR-iCre^Tg/+^, ROSA26-hGata2^Tg/+^, *Actb^−/−^* embryos (*Actb^−/−^* R26+hGata2^EpoR-iCre/+^, breeding scheme in Figure 6A). Unlike their *Actb^−/−^* embryo littermates we could obtain viable normal looking *Actb^−/−^* R26+hGata2^EpoR-iCre/+^ embryos until E11.5, providing strong genetic evidence that erythroid-restricted transgene expression of *Gata2* is capable of rescuing the lethality of the *Actb^−/−^* embryos at E10.5. However, despite this partial rescue all *Actb^−/−^* R26+hGata2^EpoR-iCre/+^ embryos were not surviving past E12.5 (Figure 6B). Percentages of surviving embryos are summarized in Table S3. Figure 6B clearly illustrates that *Actb^−/−^* R26+hGata2^EpoR-iCre/+^ embryos passed the primitive erythropoiesis phase and show prominent RBCs filled blood vessels at E11.5. We observed some heterogeneity in the rescue abilities of the embryos at E11.5 (Figure 6C). However, we found a direct correlation between the degree of normality and the overall *Gata2* expression levels, with the most normal looking rescue embryos also expressing higher *Gata2* levels (corresponding to both endogenous and transgene transcripts). qRT-PCR experiments on the yolk sacs of *Actb^−/−^* R26+hGata2^EpoR-iCre/+^ embryos demonstrate that the rescue embryos displaying higher *Gata2* values (Figure 7A–C, light-grey bars) also have higher *Gata1*, Hbb-y and Hbb-bh1 expression levels at both E10.25 and E11.5 (Figure 7A,C). *Gata2* target genes show variable expression profiles in the rescue embryos (Figure 7B). Interestingly, expression levels of gamma-actin did not decrease in the rescue embryos at E10.25 (Figure 7B) and at E11.5 (data not shown).

## Discussion

This study has revealed an unexpected role for beta-actin during development. Beta-actin has been described in protrusive cellular structures and has traditionally been associated with a major role in cell migration [6], [8], [38]. The *Actb^−/−^* embryos appear normal until E8.5 and they undergo gastrulation without any obvious defects. Cell migration processes at these developmental stages therefore seem to be intact. We investigated other events requiring cell migration processes such as formation of the vasculature and hematopoietic system in the embryo. PECAM-1 staining suggests some vessel disorganization in the embryo proper and we also found vascular defects in the yolk sac. This could indicate a cell migration defect of hemangioblast cells, a progenitor population with both hematopoietic and vascular potential. These cells are thought to migrate from the embryo proper to the blood islands in the yolk sac where cells differentiate into primitive erythrocytes and endothelial cells. However, by tracking hemangioblast cells expressing LacZ under the *Flk1* promotor [39], [40], we observed no dramatic block in migration of hemangioblast cells at E9.5. This strongly suggests that a cell migration problem of hemangioblast cells per se is not causing the hematopoietic problems in the embryo and consequently has no major contribution to the observed lethality.

Our results however strongly suggest that an erythroid differentiation defect is most likely responsible for the observed embryonic lethality at E10.5 and one of the putative downstream players involved is *Gata2*. Indeed, the expression of this key erythroid transcription factor was dramatically downregulated at E8.5. Similar to the *Actb^−/−^* embryos in this study, *Gata2* knockout embryos show marked anemia and fail to survive beyond the stage of primitive hematopoiesis [41]. Transgenic expression of *Gata2* specifically in erythroid cells rescued the embryos from lethality at E10.5. The *Actb^−/−^* R26+hGata2^EpoR-iCre/+^ embryos displayed obvious remodeled RBC containing blood vessels in the yolk sac and embryo proper and the slight variations amongst those embryos could be correlated with the levels of transgenic *Gata2* expression. After rescue the *Actb^−/−^* R26+hGata2^EpoR-iCre/+^ embryos displayed similar levels of embryonic hemoglobins Hbb-y and Hbb-bh1, in line with restoration of primitive erythropoiesis. The vasculature of yolk sac and embryo proper appears intact at E11.5 in *Actb^−/−^* R26+hGata2^EpoR-iCre/+^ embryos. This highly suggests that the vascular malformations observed in the *Actb^−/−^* embryo proper immediately before lethality and the vascular remodeling defects in the yolk sac from E9.5 onwards are secondary defects, whereas downregulation of *Gata2* and the associated block in primitive erythropoiesis is the primary defect causing the observed lethality at E10.5. The lack of full genetic rescue by erythroid-restricted GATA2 transgene expression could imply important roles of *Gata2* in later stages of cardiovascular development [42], [43] that are not rescued using this approach.

One scenario whereby absence of beta-actin leads to reduced *Gata2* mRNA levels is that *Gata2* expression is modulated by beta-actin in the nucleus. The nuclear role of beta-actin has recently been consolidated and it appears to have a potent role in the regulation of transcription and gene expression by various molecular means [10], [44], [45]. Our ChIP analysis supports a function of beta-actin in the nucleus since we detected beta-actin binding to specific regions of the *Gata2* promotor. One of the amplicons that was picked up by the ChIP experiment partly overlapped with a highly conserved region in the *Gata2* promoter, suggesting the presence of important regulatory sequences in this region of the promoter. Indeed, the detailed analyses revealed multiple consensus sites for transcription factors with known roles in erythropoiesis and/or hematopoiesis. However, it is not clear whether these transcription factors bind to the *Gata2* promotor *in vivo* nor is it evident from literature that they associate with the beta-actin protein. It also remains to be clarified whether beta-actin itself binds to the promotor as a transcription factor or whether it is part of a transcriptional complex. However, taking the previously described roles of beta-actin into account [10], [11], [12], [44], [45], we favor the possibility whereby beta-actin binds the promotor of *Gata2* in complex with other proteins.

Gamma-actin is, together with beta-actin, one of the main actin isoforms during embryonic development. It is approximately 2-fold upregulated in the embryo proper and in MEFs isolated from the *Actb−/−* embryos [13]. We observed similar compensatory mechanisms in *Actb−/−* yolk sacs (Figure S1A–C). It remains to be clarified whether such an upregulation also occurs in the *Actb−/−* erythroid precursors but in two independent studies using *Actb−/−* MEFs and T-cells, an upregulation of the other cytoplasmic actin isoform has been observed [13], [18]. Therefore, in addition to beta-actin specific nuclear effects on *Gata2* expression, lack of cytoskeletal integrity which is important in erythrocytes, cannot completely be excluded.

In summary, our results reveal a novel role for beta-actin in the early phases of hematopoiesis. By fine-tuning *Gata2* levels beta-actin plays a role in primitive and definitive erythropoiesis in the early mouse embryo. This was further demonstrated by transgenic expression of *Gata2* in the erythroid lineages, as rescue embryos were able to pass the primitive erythropoiesis phase. The functions of beta-actin described here seem very specific for this actin isoform as increased expression of gamma-actin cannot rescue this phenotype. As well, transgenic expression of *Gata2* does not lead to a normalization of gamma-actin expression levels and gamma-actin appears not to associate with regions in the *Gata2* promotor that are, however, bound by beta-actin in ChIP experiments. This study has therefore shed new light on novel specific functions of the beta-actin isoform during mouse embryogenesis.

## Supporting Information

Figure S1 **Compensatory up regulation of gamma-actin occurs in Actb−/− yolk sacs.** (A) Relative gamma-actin mRNA levels measured by qRT-PCR in yolk sacs of E8.5 and E10.25 embryos. Both embryonic stages show an approximately two fold increase in gamma-actin expression. Bars represent mean ±SEM; **P<.01, ***P<.001. (B) Western Blot analysis of gamma-actin protein level in yolk sacs of E8.5 embryos. A slight increase of gamma-actin can be detected in Actb−/− yolk sacs versus Actb+/+ yolk sacs. (C) Western Blot analysis of gamma-actin protein level in yolk sacs of E10.25 embryos. The increase of gamma-actin protein level in Actb−/− yolk sacs is more pronounced. (D) Comparison of expression of mouse beta- and gamma actin at gastrula and organogenesis stage, data are from http://www.ncbi.nlm.nih.gov/unigene.
(TIFF)

Figure S2 **Consensus sites possibly important for erythropoiesis in amplicon 3 and 8.** Detail analysis of the large amplicon 3 (amplicon 3+ regions between amplicon 2–3 and 3–4) and amplicon 8 reveal multiple DNA consensus sites for binding of transcription factors possible involved in erythropoiesis in mouse.
(TIFF)

Table S1 **Overview of the primer sets used in this study.**
(TIFF)

Table S2 **Overview of the primer sets used for the ChIP analysis and gene location of the corresponding amplicon.**
(TIFF)

Table S3 **Percentages of surviving embryos.** Mendelian ratios are given in bold under the genotype.
(TIFF)
