# Supplementary figures and images for: Beta-Actin Is Involved in Modulating Erythropoiesis during Development by Fine-Tuning Gata2 Expression Levels
Source: PLoS One. 2013 Jun 26;8(6):e67855. doi: 10.1371/journal.pone.0067855 (PMC3694046; doi:10.1371/journal.pone.0067855)

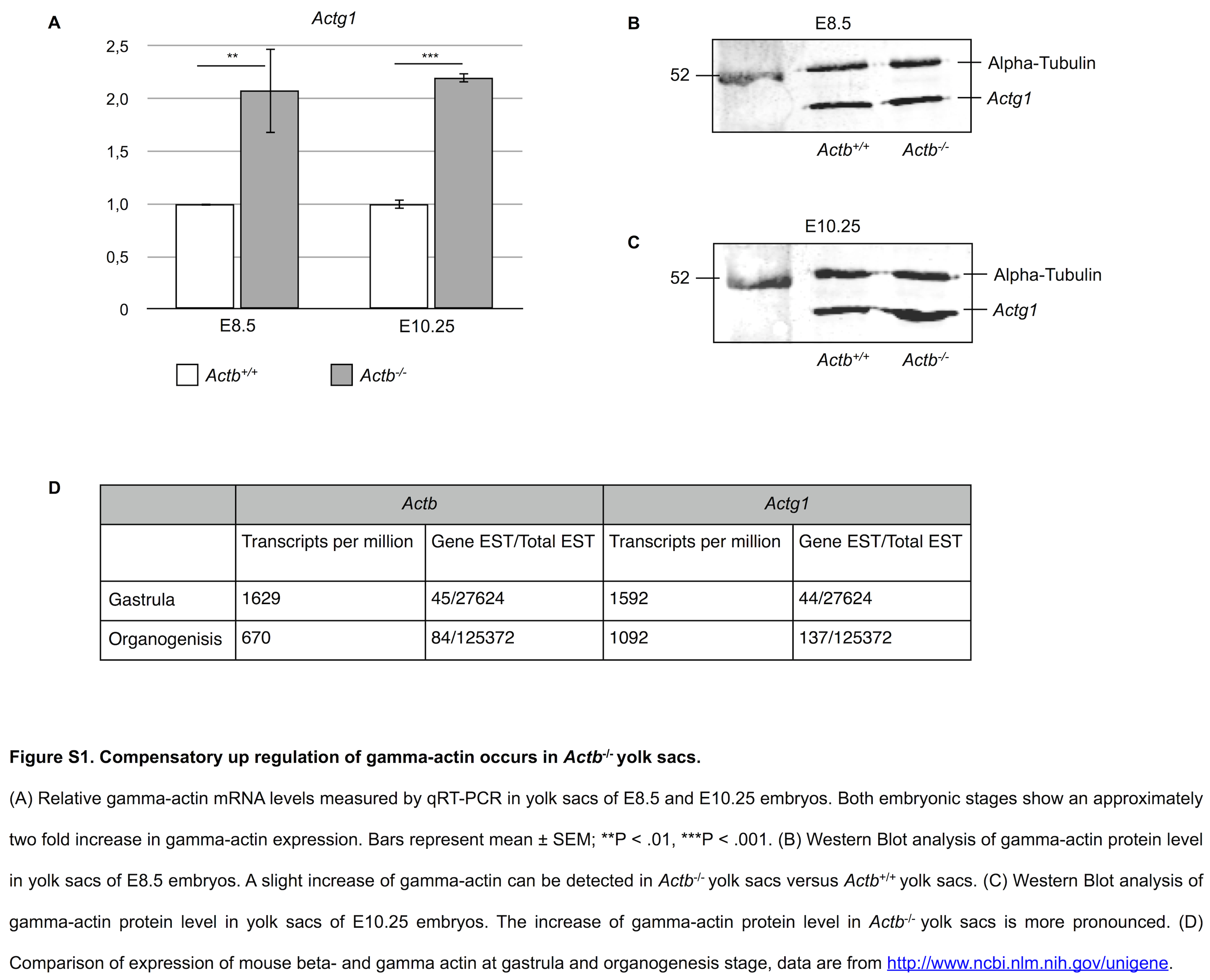

Supplement: Figure S1 — Compensatory up regulation of gamma-actin occurs in Actb−/− yolk sacs. (A) Relative gamma-actin mRNA levels measured by qRT-PCR in yolk sacs of E8.5 and E10.25 embryos. Both embryonic stages show an approximately two fold increase in gamma-actin expression. Bars represent mean ±SEM; **P<.01, ***P<.001. (B) Western Blot analysis of gamma-actin protein level in yolk sacs of E8.5 embryos. A slight increase of gamma-actin can be detected in Actb−/− yolk sacs versus Actb+/+ yolk sacs. (C) Western Blot analysis of gamma-actin protein level in yolk sacs of E10.25 embryos. The increase of gamma-actin protein level in Actb−/− yolk sacs is more pronounced. (D) Comparison of expression of mouse beta- and gamma actin at gastrula and organogenesis stage, data are from http://www.ncbi.nlm.nih.gov/unigene. (TIFF) [file pone.0067855.s001.tiff]

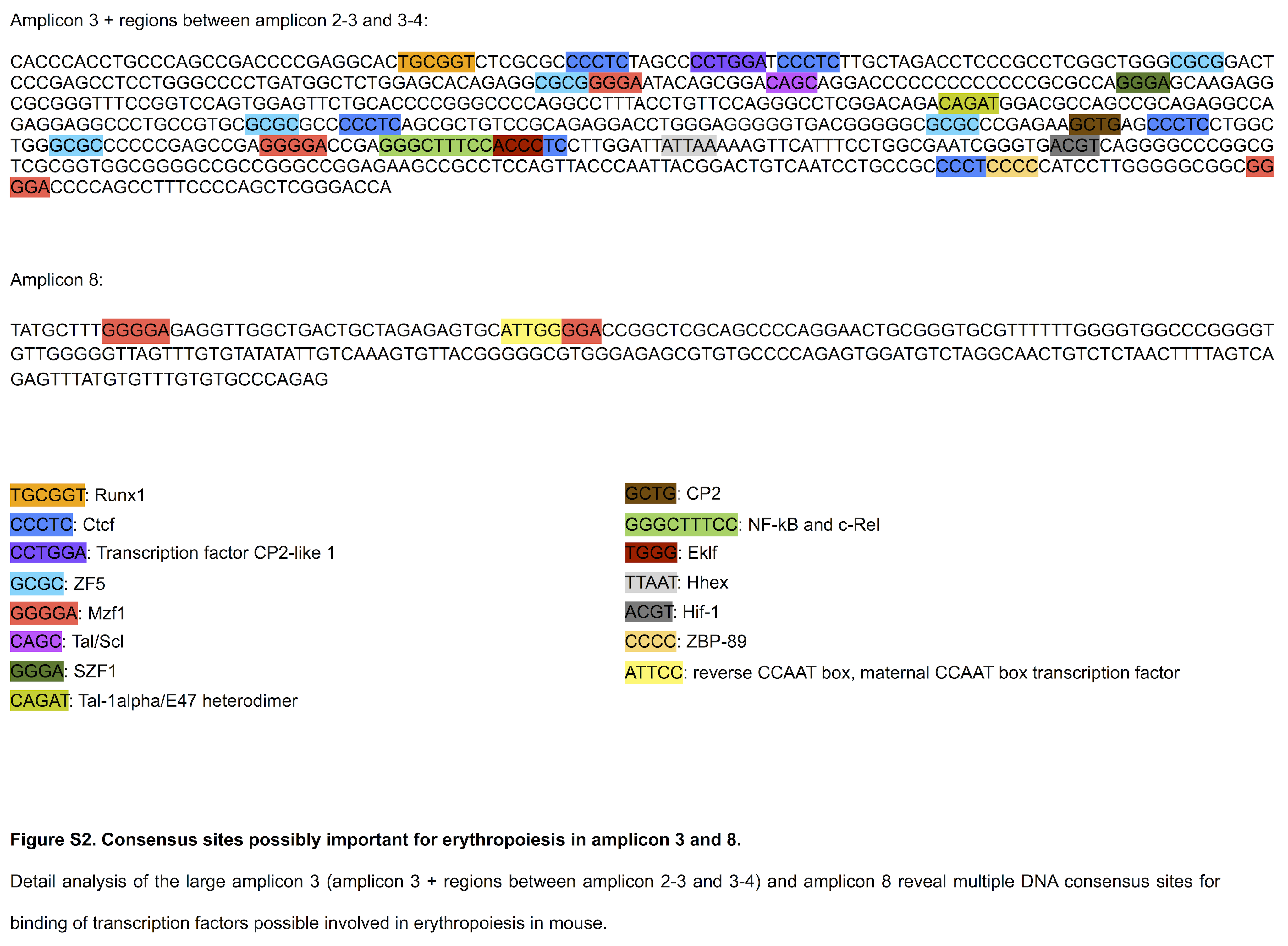

Supplement: Figure S2 — Consensus sites possibly important for erythropoiesis in amplicon 3 and 8. Detail analysis of the large amplicon 3 (amplicon 3+ regions between amplicon 2–3 and 3–4) and amplicon 8 reveal multiple DNA consensus sites for binding of transcription factors possible involved in erythropoiesis in mouse. (TIFF) [file pone.0067855.s002.tiff]

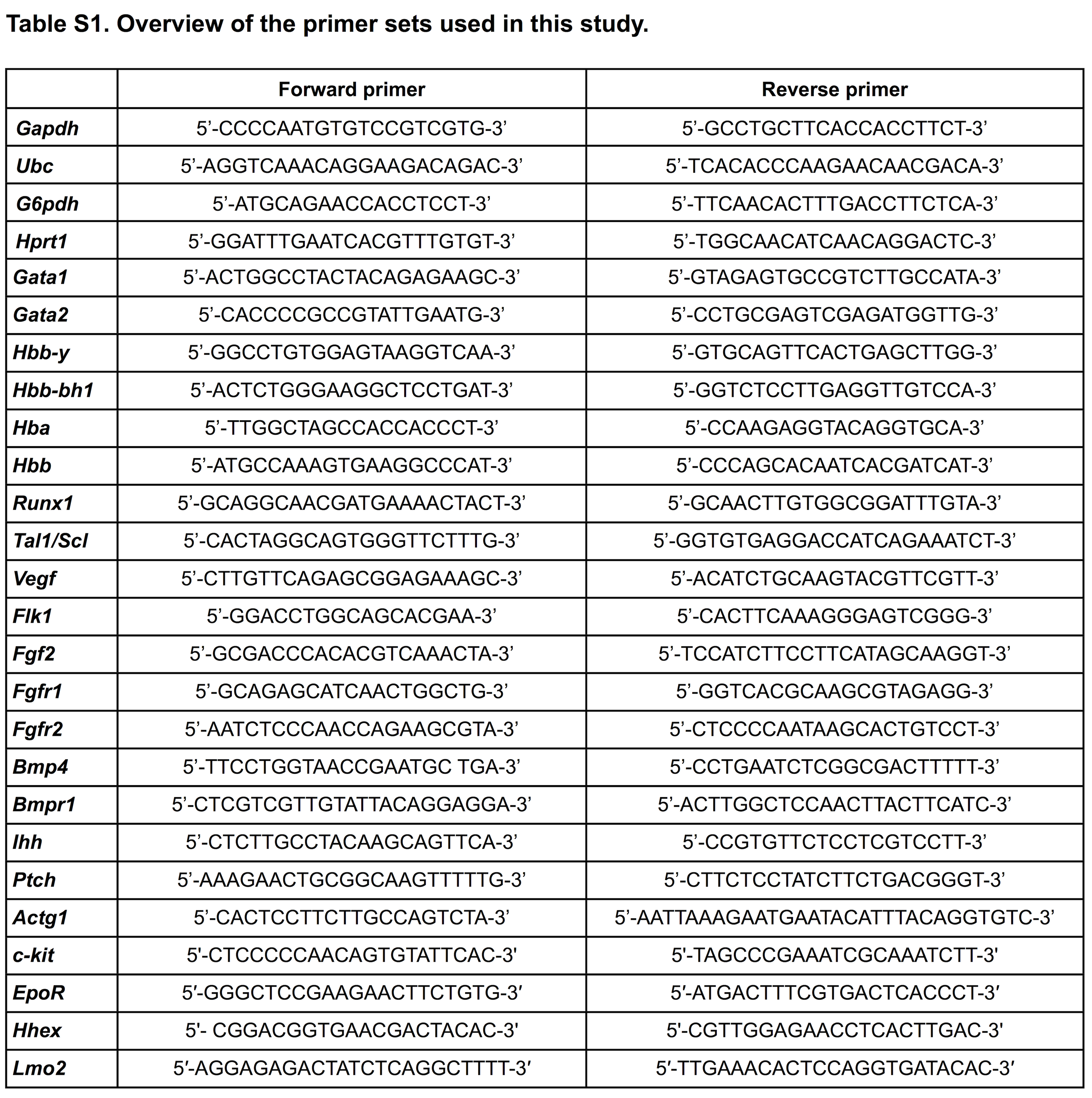

Supplement: Table S1 — Overview of the primer sets used in this study. (TIFF) [file pone.0067855.s003.tiff]

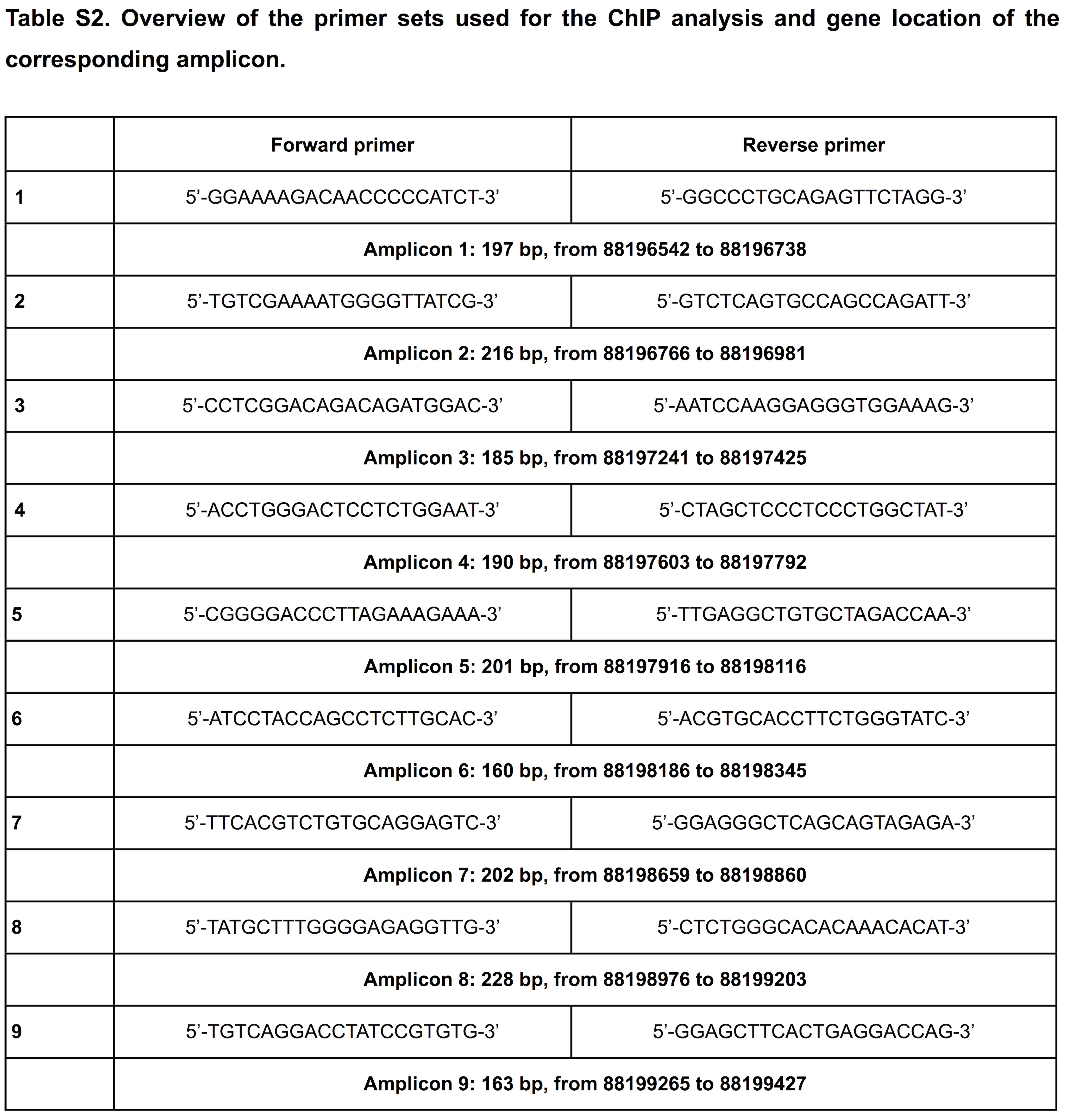

Supplement: Table S2 — Overview of the primer sets used for the ChIP analysis and gene location of the corresponding amplicon. (TIFF) [file pone.0067855.s004.tiff]

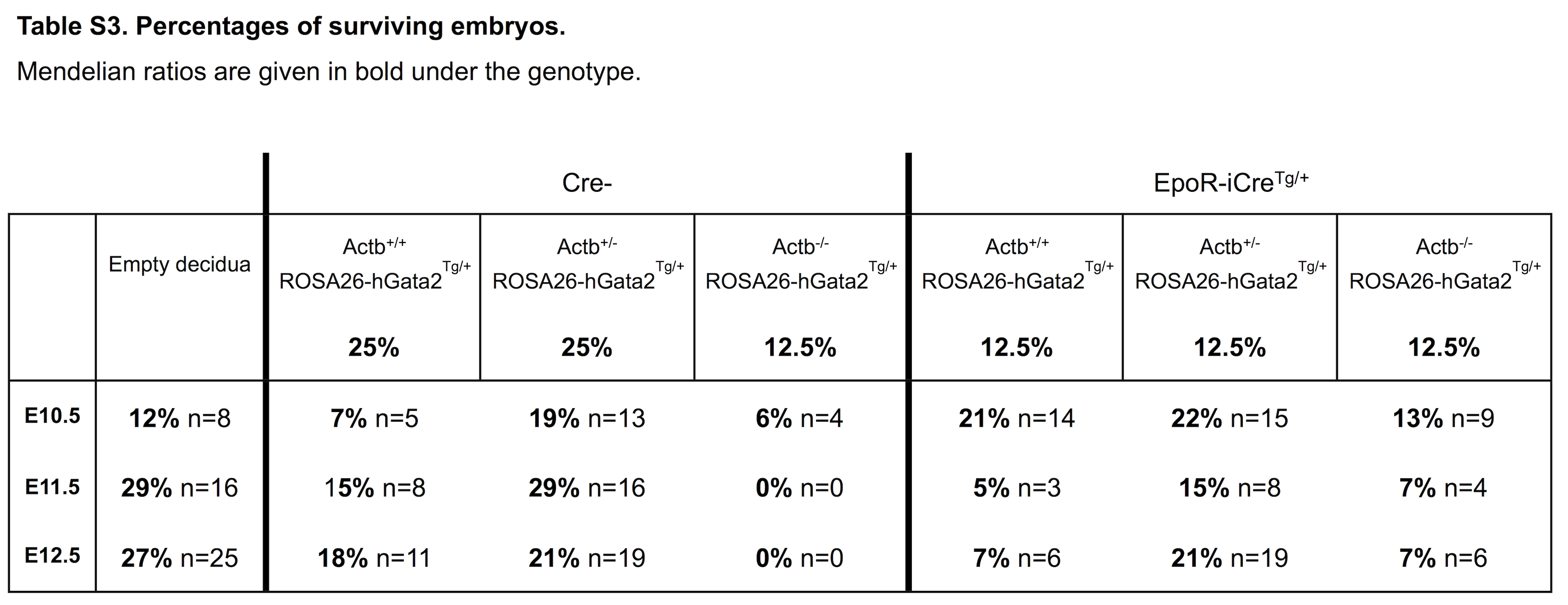

Supplement: Table S3 — Percentages of surviving embryos. Mendelian ratios are given in bold under the genotype. (TIFF) [file pone.0067855.s005.tiff]
